# Expression and allele frequencies of Thymic stromal lymphopoietin are a key factor of breast cancer risk

**DOI:** 10.1002/mgg3.813

**Published:** 2019-06-17

**Authors:** Abdelhabib Semlali, Mikhlid Almutairi, Narasimha Reddy Parine, Abdullah Al Amri, Rafa Almeer, Mohammad S. Alanazi, Mahmoud Rouabhia

**Affiliations:** ^1^ Groupe de Recherche en Écologie Buccale, Faculté de Médecine Dentaire Université Laval Québec Canada; ^2^ Department of Biochemistry College of Science King Saud University Riyadh Kingdom of Saudi Arabia; ^3^ Zoology Department College of Science King Saud University Riyadh Kingdom of Saudi Arabia

**Keywords:** breast cancer, gene expression, polymorphism, TSLP

## Abstract

**Background:**

Thymic stromal Lymphopoeitin (TSLP) is a key cytokine involved in inflammation and cancer progression. TSLP gene polymorphisms have been associated with increased susceptibility to cancer progression in different organs. We performed a control case study to examine the correlation of expression and polymorphisms of three nucleotides in TSLP with breast cancer (BC) risk in Saudi Arabian females.

**Materials and methods:**

The study was conducted on 116 healthy control subjects and 127 female patients with BC for the purpose of genotyping. Ten matching tissues provided data on immunohistochemistry to evaluate TSLP expression. Three SNPs (rs10043985, rs2289276, and rs3806933) were genotyped with TaqMan allelic discrimination assay. The patients' ages and estrogen receptor statuses were used to investigate the potential correlations between the different variations of TSLP genotypes and BC risk.

**Results:**

BC tissues expressed positive immuno‐staining for TSLP at a high rate compared to normal matching breast tissues. Malignant breast tumors exhibited higher TSLP expression than benign breast tumors. We also found that the *rs3806933* (T) allele frequency decreased the risk of developing BC in the study population (OR = 0.356, *p* = 0.00027) significantly (0.356 times). Interestingly, statistical analysis revealed that the genotype mutant (AC) and the allele mutant (C) of rs10043985 within TSLP were significantly correlated with an increased BC risk (odds ratio [OR] = 4.762, confidence interval [CI] = 1.000–22.666, *p* = 0.03244; OR = 4.762, CI = 1.000–22.666, *p* = 0.03244; and OR = 4.575, CI = 0.975–21.464, *p* = 0.03516, respectively). In addition, the AC and AC + CC genotypes of TSLP rs10043985 were confirmed to be associated with an increased risk of BC risk in women aged above 48 years, compared with the AA genotype (AC and AC + CC vs. AA: OR = 9.468, CI = 0.493–181.768, *p* = 0.04537).

**Conclusion:**

The results reveal significant correlation between SNPs in TSLP and BC progression in Saudi Arabian female patients.

## 1 INTRODUCTION

Worldwide, breast cancer (BC) is the predominant type of cancer in women (Siegel, Miller, & Jemal, 2016), and it is the leading cause of cancer mortality among women in the Kingdom of Saudi Arabia (KSA). BC accounts for approximately 24% of all newly diagnosed malignancies in Saudi females every year (Al‐Amoudi et al., 2016). Furthermore, it is more commonly diagnosed at an advanced stage in Saudi female patients than in women patients in Western countries (Al‐Khamis, 2018). One promising way to reduce the mortality and morbidity of BC in Saudi females is to discover new diagnostic markers for its early diagnosis.

The etiology and development of BC are caused by a multi‐step process involving a combination of several factors, including hormonal, genetic, and environmental factors (Key, Verkasalo, & Banks, 2001). In the present study, we focus on the genetic variation factors involved in immune inflammation and their role in the progression of BC. Accumulating evidence shows that inflammation plays a critical role in cancer initiation and progression (Grivennikov, Greten, & Karin, 2010). Currently, epidemiological studies report that more than 25% of all cancers are directly associated with chronic infections and diverse kinds of unresolved inflammation (Vendramini‐Costa & Carvalho, 2012). However, chronic inflammation has been known to play a critical role in cancer pathogenesis, including BC (Coussens & Werb, 2002; Mantovani, Romero, Palucka, & Marincola, 2008).

TSLP is an interleukin (IL)‐7‐like cytokine named ‘“master switch,”’ generally expressed by epithelial cells and capable of promoting a Th2‐mediated inflammatory immune response (Guillot‐Delost et al.,; Liu et al., 2007; Soumelis et al., 2002; Ziegler, 2010). Although marine and human TSLP shows only 43% amino acid identity, it performs similar functions in both species (Liu et al., 2007; Reche et al., 2001). In addition, TSLP plays an important role in activating immune cells, such as B‐cells and dendritic cells (DCs) (Ziegler, 2010). More evidence has reported an essential role of TSLP in the development of allergic disorders, including atopic dermatitis and allergic bronchial asthma (Sebastian, Borowski, Kuepper, & Friedrich, 2008; Ziegler, 2010) as well as genesis of malignant tumors (Olkhanud et al., 2011). An increased TSLP level was detected in several tumors, including malignant breast and pancreatic tumors, both connected with T helper (Th) 2‐related chronic inflammation (Olkhanud et al., 2011; Pedroza‐Gonzalez et al., 2011). A recent study also found a significant decrease of TSLP in patients with colon cancer compared to tissues surrounding a tumor, as well as a negative association between TSLP levels and the score of clinical staging of colon cancer (Yue et al., 2016). In addition, Watanabe, Saito, Miyatani, Ikeguchi, and Umekita (2015) have clearly reported that, a high level of TSLP expression indicates poor prognosis in patients with gastric cancer (Watanabe et al., 2015). Many studies have indicated that different types of cells other than epithelial cells and epidermal keratinocytes express TSLP. These cells include airway smooth muscle cells, mast cells, dendritic cells, trophoblasts, fibroblasts, and cancer cells. The production of TSLP can be triggered by various environmental factors, such as toll‐like receptor ligands, viruses, diesel exhaust, microbes, helminths, allergen sources, chemicals, and cigarette smoke (Takai, 2012).

A recent study suggested that genetic polymorphisms in TSLP were significantly associated with BC prognosis among Korean women (Choi et al., 2014). In the current study, we investigated the TSLP polymorphisms and TSLP expression in female Saudi BC patients.

## 2 MATERIALS AND METHOD

### 2.1 Ethics statement and consent

All breast tissues and bloods samples for the current study were collected by Abdulrahman Al Naeem, department of Women's Imaging, King Fahad Medical City, Riyadh, Saudi Arabia. and by Doctor Sana Abdulla Ajaj from Family Medicine department, College of Medicine, King Saud University, Riyadh, Saudi Arabia. The study design was approved by the local Ethic Committee of King Faisal Medical city (local ethics committee number 15‐089E). All cases and controls agreed to participate by signing an informed‐consent document. Clinical data including age, sex, family history and medical conditions, was completed by all participants. Determination of the estrogen receptor (ER) was also recorded through immunohistochemical analysis by Dr. Maha Arafah at King Khalid University Hospital in Riyadh, in the Kingdom of Saudi Arabia.

### 2.2 Control case study

This study includes 127 Saudi women diagnosed with histologically confirmed BC at King Faisal Medical City in Riyadh from 2012 to 2016 prior to any medical treatment. The controls consisted of 116 blood samples isolated from normal women who did not have any BC clinical signs. Then, genomic DNA was extracted from all blood samples, which were obtained from the case and control groups and successfully genotyped. The matching tissue samples were used for determining the protein level via immunohistochemistry (IHC).

The patients and controls were matched within five years by age and sex. The eligibility criteria for the controls were no history of previous cancer and normal mammography results. Demographic data including age (≤48 and >48), family history of BC, smoking and alcohol behavior, and clinical pathological data (such as estrogen receptor status [ER] and progesterone receptor status [PR]) are listed in Table 1.

**Table 1:** Selected clinical characteristics of Female Saudi patients with BC and the normal controls

Variable	Character	Patients *n* (%)	Controls *n* (%)
Total persons	–	127 (100%)	116 (100%)
Age (Years)	≤48	45 (35.43%)	62 (53.45%)
Median age (48 ± 8.2)	>48	82 (64.57%)	54 (46.55%)
Estrogen receptor	ER+	76 (59.84%)	–
	ER−	49 (38.58%)	–
	Undetermined	2 (1.57%)	
Progesterone receptor	PR+	71 (55.91%)	–
	PR−	55 (43.31%)	–
	Undetermined	1 (0.79%)	
HER Status	HER+	49 (38.58%)	–
	HER−	78 (61.42%)	–

### 2.3 Immunohistochemistry assay

The paraffinized biopsied tissues were used to investigate the TSLP protein level via IHC technique as described previously by Semlali et al. (2015), Semlali, Jacques, Koussih, Gounni, and Chakir (2010), Semlali et al. (2017), Semlali et al. (2016). After being deparaffinized, rehydrated, and incubated for 30 min in a hot air oven at 60°C, the slides were processed in an automated IHC slide staining system (the Benchmark XT from Ventana Medical Systems Inc., [USA]) and incubated a second time with anti‐human (anti‐TSLP) primary antibodies (1:100; Santa Cruz Biotechnology, CA) for 1 hr at 37°C. The ultra‐view multimer detection system from Ventana (Ventana Medical Systems Inc., USA) was used to conduct the examination. The immunolocalized TSLP protein was visualized using a copper‐enhanced DAB reaction. Slides were then stained using Hematoxylin II (Ventana Medical Systems Inc., USA), and incubated in bluing reagent for 4 min. Finally, coverslips were applied.

### 2.4 Genomic DNA preparation

All study participants provided 2–3 ml of blood, which was collected in vials containing 5% EDTA. Genomic DNA extractions were performed using 200 μl of whole blood according to the manufacturer's protocol through a QIAamp DNA blood mini kit (Qiagen, Valencia, CA). The DNA samples were stored at −20°C until they were used. The concentration of extracted genomic DNA was calculated with a Nano‐Drop 8000 spectrophotometer (Thermo Scientific, USA), and DNA purity was evaluated by calculating the absorbance ratios of A260/A280 and A260/A230.

### 2.5 SNP selection and genotyping methods

A total of three polymorphisms were investigated and genotyped with a TaqMan allelic discrimination assay, as reported previously (Alanazi et al., 2013). The SNPs were selected on the basis of their positions and frequencies, as well as their associations with cancer risk as described previously. Sample genotyping was performed in a 96‐well format with the ABI 7500 real‐time PCR instrument (Applied Biosystems, Foster City, CA). The polymorphisms that were studied for TSLP were rs10043985 (A/C), rs2289276 (C/T), and rs3806933 (C/T). These SNPs are located in the promoter, 5′‐untranslated region (UTR), and promoter, respectively. PCR amplifications were performed in an overall volume of 10 μL/reaction mixture, containing 10–20 ng of DNA, 5.6 μL of 2X TaqMan Universal Master Mix, and 200 nm of each primer. The sequences of the primers and probe mixtures used in this study were purchased from the Applied Biosystems Company (ThermoFisher, Grand Island, NY, USA). Five percent of the samples was randomly selected and subjected to repeat analysis to confirm genotyping quality.

### 2.6 Statistical methods

The genotype and allele frequencies obtained were analyzed with Hardy–Weinberg equilibrium (http://ihg2.helmholtz-muenchen.de/cgi-bin/hw/hwa1.pl) (HWE). Chi‐square tests and allelic odds ratios (ORs) were used to compare genetic variables between the patients and the control group. A 95% confidence interval (CI) was calculated for each allele and genotype using Fisher's exact test (two‐tailed p values) to study the association between TSLP polymorphisms and BC risk. Statistical analysis was performed using SPSS version 16.0 statistical software (SPSS, Chicago, IL). All *p* values less than 0.05 indicated statistical significance.

## 3 RESULTS

### 3.1 Analysis of the clinical parameters of the BC cases and the controls

Table 1 represents the overall demographic and clinical characteristics of the selected population. Cases and controls did not vary substantially in demographic characteristics, but some key differences were found for other factors. The mean age of the healthy controls and the BC patients (48 ± 8.2) did not show any statistically significant differences. There were a total of 127 cases compared with 116 healthy controls, and the age of 45 patients in the cases was ≤48, whereas 82 persons was >48. By contrast, the age of the healthy controls was ≤48 for 62 persons and >48 for 54 persons. Other clinical characteristics of these populations were also obtained, such as age and nationality, family history, stage of BC, smoking habits, medications, presence of other diseases, and ER and human epidermal growth factor receptor statuses (Table 1).

### 3.2 Increased TSLP protein levels in BC tissues compared to normal breast tissues

**Figure 1:**
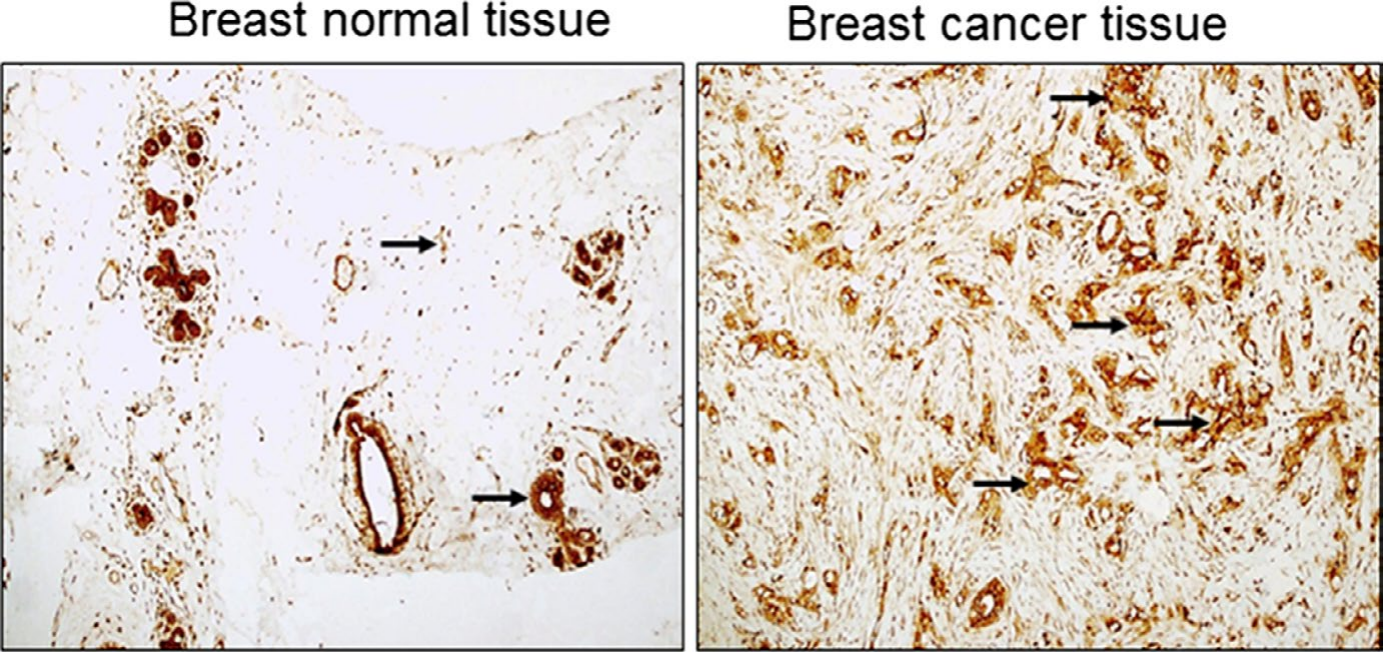
Thymic stromal lympho‐protein (TSLP) protein expression in breast cancer and normal matching tissues. Immunohistochemistry was performed by using specific TSLP antibodies (Panel 1A) *n* = 10. TSLP‐ positive staining was estimated and presented in (Panel) as follows B: 0 points, no positive color; 1 point, <20% positive staining; 2 points, 21%–50% positive staining; 3 points, 51%–75% positive staining; and 4 points, >75% positive staining. This is presented in Panel B

We used IHC to examine the difference in protein levels of TSLP in BC tissues compared to normal breast tissues. Figure 1 shows that BC tissues presented positive immuno‐staining for TSLP at high rates compared to normal breast tissues (*n* = 10). TSLP protein levels increased depending on the type of BC. In addition, malignant or cancerous tumors presented higher levels of TSLP than benign breast tumors (Figure 1).

### 3.3 Association of TSLP gene polymorphisms with the risk of BC in the Saudi population

Three TSLP SNPs (*rs3806933 C/T, rs2289276 C/T, and rs10043985 A/C*) located in the promoter region and in 5'UTR were genotyped and assessed for HWE in the present study. The genotype distributions of TSLP SNPs among BC patients and the controls are shown in Table 2. We found that the *rs3806933* (T) allele frequency significantly decreases (0.356 times) the risk of developing BC (protect effect) in the study population (OR = 0.356, *p* = 0.00027). Interestingly, we also found that the (AC) and (AC + CC) genotypes and the (C) allele of rs10043985 within TSLP significantly correlated with an increased BC risk by more than 4.5 times (OR = 4.762, CI = 1.000–22.666, *p* = 0.03244; OR = 4.762, CI = 1.000–22.666, *p* = 0.03244; and OR = 4.575, CI = 0.975–21.464, *p* = 0.03516, respectively, as indicated by Table 2. However, the distribution of allele frequencies for TSLP rs2289276 was similar between BC patients and healthy controls in the Saudi population (Table 2).

**Table 2:** Distribution of genotype and allele frequencies of TSLP gene SNPs in Female Saudi patients with BC and the normal controls

SNP	Genotype/allele	Breast	Control	OR	95% CI	*χ* ^2^	*p* value
rs10043985	**Total**	**95**	**93**				
	AA	86 (0.91)	91 (0.98)	Ref			
	AC	9 (0.09)	2 (0.02)	4.762	1.000–22.666	4.58	0.03244*
	CC	0 (0.00)	0 (0.00)	1.058	0.021–53.90	nan	1.000
	AC + CC	9 (0.09)	2 (0.02)	4.762	1.000–22.666	4.58	0.03244*
	A	181 (0.95)	184 (0.99)	Ref			
	C	9 (0.05)	2 (0.01)	4.575	0.975–21.464	4.44	0.03516*
rs2289276	**Total**	**158**	**176**				
	CC	74 (0.47)	77 (0.44)	Ref			
	CT	60 (0.38)	85 (0.48)	0.7345	0.46397–1.16277	1.73684	0.186061
	TT	24 (0.15)	14 (0.08)	1.7838	0.85764–3.71003	2.43526	0.108448
	CT + TT	84 (53)	99 (0.56)	0.8829	0.57336–1.35949	0.31998	0.571550
	C	208 (0.66)	239 (0.68)	Ref			
	T	108 (0.34)	113 (0.32)	1.0982	0.79534–1.51638	0.32383	0.5695
rs3806933	**Total**	**95**	**184**				
	CC	0 (0.00)	1 (0.01)	Ref			
	CT	30 (0.32)	21 (0.11)	4.256	0.165–109.523	1.39	0.23834
	TT	65 (0.68)	162 (0.88)	1.209	0.049–30.067	0.40	0.52682
	CT + TT	95 (1.0)	183 (0.99)	1.561	0.063–38.694	0.52	0.47163
	C	30 (0.16)	23 (0.06)	Ref			
	T	160 (0.84)	345 (0.94)	0.356	0.200–0.632	13.27	0.00027**

Note

Values in bold represent significant results.

Abbreviation: Ref, Reference allele.

* *p* < 0.05.

** *p* < 0.005.

### 3.4 Genotype and allele frequencies distribution of TSLP and their association with different clinical parameters

#### 3.4.1 Variation of genotype and allele frequencies of TSLP based on age at BC diagnosis

The frequency distributions of the TSLP genotypes were analyzed according to patient age. This study demonstrated that the average age of onset of the examined patients with BC was 48 years of age. We further examined the correlation between three TSLP SNPs with age of BC at diagnosis by dividing the patients into the following groups: aged less than 48 years (*n* = 45) or more than 48 years (*n* = 82) years. This study demonstrated that the T allele of the TSLP SNP rs3806933 showed a significant association with protected risk of BC in patients who were diagnosed with BC at an age below 48 years (OR = 0.368, CI = 0.172–0.785, *p* = 0.007) or above 48 years (OR = 0.350, CI = 0.145–0.846, *p* = 0.015) (Table 3A, 3). Additionally, the AC and AC + CC genotypes of TSLP rs10043985 were associated with an increased risk of BC in women above 48 years by more than nine times (OR = 9.46, *p* = 0.045), but not in women aged below 48 years (*p* < 0.05) (Tables 3A, 3). However, in the Saudi population, the distribution of genotype and allele frequencies for rs2289276 was similar between women with BC and healthy women independent of a BC diagnosis in both groups of age.

**Table 3:** Genotype and allele frequencies of TSLP gene SNPs in Female Saudi patients with BC and normal controls based on age at BC diagnosis

SNP	Genotype/allele	Breast	Control	OR	95% CI	*χ* ^2^	*p* value
Aged below 48 years							
rs10043985	**Total**	**53**	**53**				
	AA	48 (0.91)	51 (0.96)	Ref			
	AC	5 (0.09)	2 (0.04)	2.656	0.492–14.346	1.38	0.24068
	CC	0 (0.00)	0 (0.00)	1.062	0.021–54.572	nan	1.000
	AC + CC	5 (0.09)	2 (0.04)	2.656	0.492–14.346	1.38	0.24068
	A	101 (0.95)	104 (0.98)	Ref			
	C	5 (0.05)	2 (0.02)	2.574	0.488–13.573	1.33	0.44614
rs2289276	**Total**	**74**	**88**				
	CC	32 (0.43)	35 (0.40)	Ref			
	CT	30 (0.41)	45 (0.51)	0.72917	0.37471–1.41893	0.866562	0.350871
	TT	12 (0.16)	8 (0.09)	1.64063	0.59459–4.5269	0.922956	0.328989
	CT + TT	42 (0.57)	53 (60)	0.86675	0.46278–1.62335	0.199627	0.655138
	C	94 (0.64)	115 (0.65)	Ref			
	T	54 (0.36)	61 (0.35)	1.08301	0.68609–1.70958	0.117257	0.7322
rs3806933	**Total**	**52**	**91**				
	CC	0 (0.00)	1 (0.01)	Ref			
	CT	18 (0.35)	11 (0.12)	4.826	0.18–128.789	1.55	0.21288
	TT	34 (0.65)	79 (0.87)	1.302	0.052–32.762	0.43	0.51260
	CT + TT	52 (1.0)	90 (0.99)	1.740	0.070–43.497	0.58	0.44810
	C	18 (0.17)	13 (0.07)	Ref			
	T	86 (0.83)	169 (0.93)	0.368	0.172–0.785	7.08	0.00781*
Aged above 48 years							
rs10043985	**Total**	**42**	**40**				
	AA	38 (0.90)	40 (1.0)	Ref			
	AC	4 (0.10)	0 (0.00)	9.468	0.493–181.768	4.00	0.04537*
	CC	0 (0.00)	0 (0.00)	1.052	0.020–54.343	nan	1.000
	AC + CC	4 (0.10)	0 (0.00)	9.468	0.493–181.768	4.00	0.04537*
	A	80 (0.95)	80 (1.0)	Ref			
	C	4 (0.05)	0 (0.00)				
rs2289276	**Total**	**84**	**88**				
	CC	42 (0.50)	42 (0.48)	Ref			
	CT	30 (0.36)	40 (0.45)	0.75	0.39627–1.4195	0.78252	0.374683
	TT	12 (0.14)	6 (0.07)	2	0.68652–5.82649	1.652778	0.177718
	CT + TT	42 (0.50)	46 (0.52)	0.91304348	0.50196–1.66078	0.088843	0.7656
	C	114 (0.68)	124 (0.70)	Ref			
	T	54 (0.32)	52 (0.30)	1.12955466	0.71455–1.78559	0.272005	0.6019
rs3806933	**Total**	**43**	**93**				
	CC	0 (0.00)	0 (0.00)	Ref			
	CT	12 (0.28)	10 (0.11)	1.190	0.022–65.315	nan	1.0000
	TT	31 (0.72)	83 (0.89)	0.377	0.007–19.423	nan	1.0000
	CT + TT	43 (1.0)	93 (1.0)	0.465	0.009–23.836	nan	1.0000
	C	12 (0.14)	10 (0.05)	Ref			
	T	74 (0.86)	176 (0.95)	0.350	0.145–0.846	5.82	0.01585*

Note

Values in bold represent significant results.

Abbreviation: Ref, Reference allele.

* *p* < 0.05.

#### 3.4.2 Variation of genotype and allele frequencies of TSLP based on ER status

We analyzed the associations between SNPs in TSLP and patient clinicopathological features, including ER status. The results for TSLP SNP rs3806933 show that compared with the C allele, the T allele in patients with ER‐positive BC and ER‐negative BC was associated with BC risk protection (OR = 0.418, CI = 0.195–0.897; *p* = 0.02169 in ER+ve and OR = 0.341, CI = 0.175–0.666; *p* = 0.0011 in ER‐ve). Additionally, both groups demonstrated similar distribution of the genotype frequencies (ER+ and ER‐) (Table 4A, 4). Conversely, rs10043985 increases the BC risk by more than 4.8 times, but only in the ER‐ population (OR = 4.84 and *p* = 0.0443) (Table 4B). However, no significant differences were found between the ER+ve and ER‐ve features in the cases and the controls (*p* > 0.05) in TSLP rs2289276 (Table 4A, 4).

**Table 4:** Genotype and allele frequencies of TSLP gene SNPs in Female Saudi patients with BC and the normal controls based on the status of Estrogen Receptor

SNP	Genotype/allele	Breast	Control	OR	95% CI	*χ* ^2^	*p* value
Estrogen positive							
rs10043985	**Total**	**40**	**93**				
	AA	37 (0.93)	91 (0.98)	Ref			
	AC	3 (0.07)	2 (0.02)	3.689	0.592–22.98	2.21	0.13692
	CC	0 (0.00)	0 (0.00)	2.440	0.048–125.249	nan	1.000
	AC + CC	3 (0.07)	2 (0.02)	3.689	0.592–22.98	2.21	0.13692
	A	77 (0.96)	184 (0.99)	Ref			
	C	3 (0.04)	2 (0.01)	3.584	0.587–21.877	2.17	0.24922
rs2289276	**Total**	**85**	**176**				
	CC	41 (0.48)	77 (0.44)	Ref			
	CT	34 (0.40)	85 (0.48)	0.75122	0.4337–1.30119	1.044194	0.305218
	TT	10 (0.12)	14 (0.08)	1.34146	0.54779–3.28507	0.415045	0.528221
	CT + TT	44 (0.52)	99 (0.56)	0.83469	0.49657–1.40303	0.465517	0.495752
	C	116 (0.68)	239 (0.68)	Ref			
	T	54 (0.32)	113 (0.32)	0.98459	0.66473–1.45836	0.006004	0.9382
rs3806933	**Total**	**40**	**184**				
	CC	0 (0.00)	1 (0.01)	Ref			
	CT	11 (0.28)	21 (0.11)	1.605	0.060–42.633	0.52	0.47271
	TT	29 (0.72)	162 (0.88)	0.545	0.022–13.693	0.18	0.67237
	CT + TT	40 (1.0)	183 (0.99)	0.662	0.026–16.549	0.22	0.64029
	C	11 (0.14)	23 (0.06)	Ref			
	T	69 (0.86)	345 (0.94)	0.418	0.195–0.897	5.27	0.02169*
Estrogen negative							
rs10043985	**Total**	**52**	**93**				
	AA	47 (0.90)	91 (0.98)	Ref			
	AC	5 (0.1)	2 (0.02)	4.840	0.905–25.899	4.05	0.04430*
	CC	0 (0.00)	0 (0.00)	1.926	0.038–98.610	nan	1.000
	AC + CC	5 (0.1)	2 (0.02)	4.840	0.905–25.899	4.05	0.04430*
	A	99 (0.95)	184 (0.99)	Ref			
	C	5 (0.05)	2 (0.01)	4.646	0.885–24.386	3.94	0.11327
rs2289276	**Total**	**67**	**176**				
	CC	31 (0.46)	77 (0.44)	Ref			
	CT	25 (0.37)	85 (0.48)	0.73055	0.39676–1.34516	1.019656	0.310392
	TT	11 (0.16)	14 (0.08)	1.95161	0.79904–4.76671	2.198352	0.157128
	CT + TT	36 (0.53)	99 (0.56)	0.90323	0.51331–1.58932	0.124674	0.724552
	C	87 (0.65)	239 (0.68)	Ref			
	T	47 (0.35)	113 (0.32)	1.14261	0.75114–1.7381	0.388282	0.5370
rs3806933	**Total**	**52**	**184**				
	CC	0 (0.00)	1 (0.01)	Ref			
	CT	17 (0.33)	21 (0.11)	2.442	0.094–63.751	0.79	0.37318
	TT	35 (0.67)	162 (0.88)	0.655	0.026–16.422	0.22	0.64225
	CT + TT	52 (1.0)	183 (0.99)	0.858	0.034–21.381	0.28	0.59421
	C	17 (0.16)	23 (0.06)	Ref			
	T	87 (0.84)	345 (0.94)	0.341	0.175–0.666	10.66	0.0011**

Note

Values in bold represent significant results

Abbreviations: Ref, Reference allele.

* *p* < 0.05.

** *p* < 0.005.

## 4 DISCUSSION

TSLP is a cytokine that triggers dendritic cells (DCs) to induce inflammation. Same evidences suggests their implication in allergic diseases pathogenesis and involved in many cancer progressions such as BC (Liu et al., 2007; Mayer & Dalpke, 2007; Wang, Bai, Li, Adler, & Wang, 2008; Zhang, Song, Zhao, Zhang, & Bachert, 2012). Recently, many works have suggested that TSLP can play a key role in the BC tumor microenvironment and influence BC progression. The current study is the first study interested to evaluate the protein levels of TSLP in BC tissues compared to healthy matching breast tissues and secondly to investigate the contribution of three targeting TSLP SNPs (Two in promoter region; rs3806933 and rs10043985, another located 3'UTR region; rs22892276) and their association with BC development in the Saudi female population. In the present study, we showed that, TSLP protein is significantly high expressed in BC tissues compared to matching normal tissues, suggesting possible regulation of TSLP production by Th2 inflammatory cytokines during BC progression (Nguyen, Vanichsarn, & Nadeau, 2010; Semlali et al., 2010; Ying et al., 2008, 2005). In literature, the exact role of TSLP in BC development is little studied. However, Olkhanud et al. (2011) have clearly reported that TSLP protein acts as a principal mediator of BC progression (Olkhanud et al., 2011) by promoting Th‐2 environment (Ziegler et al., 2013). However, a recent study by Ghirell et al. (2016) reports any correlation between TSLPR pathway activity and human BC progression (Ghirelli et al., 2016). On the other hand, Pedroza‐Gonzalez et al. (2011) has in turn reported that TSLP fosters human breast tumor growth by promoting type 2 inflammation (Pedroza‐Gonzalez et al., 2011). Many previous research studies support this hypothesis, reporting the key role of TSLP in promoting a tumor microenvironment through the Th‐2 cytokines expression. De Monte et al. have proven that high TSLP production as a result of pancreatic cancer is correlated with Th2 cellular infiltration (De Monte et al., 2011). Xie et al. (2013) found that TSLP secreted by cervical carcinoma cells participates in the angiogenesis of cervical cancer pathogenesis (Xie et al., 2013). Pedroza‐Gonzalez and his colleagues demonstrated the association between TSLP and Th2 microenvironment in breast tumors. Using a xeno‐transfer model, they showed that a blockade of either TSLP or OX40L induces a net reduction of tumor growth and IL‐13 secretion (Pedroza‐Gonzalez et al., 2011). Taking these papers into account, we suggest that TSLP should be considered the key player in promoting cancer development through the immune response in the tumor itself. TSLP inhibition could be an important key for the future of BC cancer therapy. Also, in this current study, we also demonstrated that, the allele frequency of the rs3806933 has a protective effect in BC development independent of age or the ER status in the study population. TSLP rs10043985 was significantly associated with an increase in BC risk only for ER‐negative Saudi women aged more than 48 years. TSLP rs10043985 increases BC risk by more than four times among BC patients relative to healthy control subjects, but TSLP rs2289276 does not present any correlation with BC susceptibility in the study population. This finding confirms previous results. Choi et al. (2014) observed an association between another SNP rs2289278 of TSLP and BC susceptibility among Korean women (Choi et al., 2014). Association studies relating phenotypes using genetic polymorphisms have been conducted in diverse populations with allergy inflammatory diseases (Ghirelli et al., 2016). In contrast, Harada et al. (2009) have clearly demonstrated that the promoter SNP rs3806933 of TSLP could create an activating protein (AP)–1 transcription factor binding site. This variant enhances AP‐1 binding to some regulatory elements, and also increases the promoter–reporter activity of TSLP (Harada et al., 2009). In a more recent study, the authors reported that TSLP gene promoter polymorphisms (rs3806933 and rs2289276) significantly increase susceptibility to asthma (Taneda et al., 2001). Overall, these findings suggest that the TSLP gene expression and TSLP polymorphism, especially promoter polymorphisms rs3806933 and rs10043985, are highly involved in the BC susceptibility of Saudi Arabian subjects.

The association between TSLP rs10043985 and an increase in BC risk only in ER‐negative Saudi woman aged more than 48 years can be explained in part by low sexual hormones such as estrogen. It is normal for estrogen to drop by the age of 48, as women approach menopause. These observations suggest that the decline in endogenous estrogen levels in postmenopausal women can be a critical risk factor for a variety of health problems including cancers. It is well documented that menopause decreases estrogen's role in inflammation. Nevertheless, Prestwood, Unson, Kulldorff, and Cushman (2004) observed that levels of proinflammatory interleukins dramatically increase during menopause in women when E2 synthesis is low (Prestwood et al., 2004).

## 5 CONCLUSION

There is increasing evidence implicating TSLP protein levels and TSLP polymorphisms in BC development. We conclude that TSLP expression and polymorphisms may be a useful prognostic marker. Targeting TSLP could have therapeutic potential. We believe that the association between these SNPs and BC risk could contribute to the development of alternative tools that may serve as biomarkers for early BC prognosis. Although the polymorphisms of these genes may be important in BC development, further research with more BC samples and additional independent studies in other ethnic populations are necessary to validate the relevance of the observed correlations.

## CONFLICTS OF INTEREST

All authors declare no conflict of interests and all authors approved the manuscript.
